# Chevalier's Instruments

**Published:** 1870-01

**Authors:** 


*Chevalier's Instruments.*—We have received from this New York firm some excavators, introducers and condensers, which, after due trial, we cannot praise too highly for temper, fineness of serrations, general form, length and finish. The introducing and condensing instruments especially, are, to say the least of them, models of their class, and we congratulate this firm on their success in producing such excellent instruments as the ones we have tested.

